# Prognostic Value of Routinely Measured Inflammatory Biomarkers in Older Cancer Patients: Pooled Analysis of Three Cohorts

**DOI:** 10.3390/cancers13246154

**Published:** 2021-12-07

**Authors:** Nadia Oubaya, Pierre Soubeyran, Nicoleta Reinald, Marianne Fonck, Mylène Allain, Sonia Zebachi, Damien Heitz, Marie Laurent, Cécile Delattre, Philippe Caillet, Jérôme Dauba, Sylvie Bastuji-Garin, Gilles Albrand, Michael Bringuier, Muriel Rainfray, Etienne Brain, Thomas Grellety, Elena Paillaud, Simone Mathoulin-Pélissier, Carine Bellera, Florence Canouï-Poitrine

**Affiliations:** 1 University Paris Est Creteil, INSERM, IMRB, F-94010 Créteil, France; nicoleta.reinald@aphp.fr (N.R.); allain.mylene@yahoo.fr (M.A.); sonia.zebachi-ext@aphp.fr (S.Z.); marie.laurent@aphp.fr (M.L.); philippe.caillet@aphp.fr (P.C.); sylvie.bastuji-garin@aphp.fr (S.B.-G.); elena.paillaud@aphp.fr (E.P.); florence.canoui-poitrine@aphp.fr (F.C.-P.); 2 Department of Public Health, Henri Mondor Hospital, AP-HP, F-94010 Créteil, France; 3 Department of Medical Oncology, Institut Bergonié, University of Bordeaux, Inserm U1218, F-33000 Bordeaux, France; p.soubeyran@bordeaux.unicancer.fr (P.S.); m.fonck@bordeaux.unicancer.fr (M.F.); t.grellety@bordeaux.unicancer.fr (T.G.); 4 Clinical Research Unit, Henri Mondor Hospital, AP-HP, F-94010 Créteil, France; 5 Oncology and Hematology Unit, Strasbourg University Hospital, Hautepierre Hospital, F-67200 Strasbourg, France; damien.heitz@chru-strasbourg.fr; 6 Department of Geriatrics, Henri Mondor Hospital, AP-HP, F-94010 Créteil, France; 7 Supportive Care Unit, Institut de Cancérologie of Lorraine Alexis Vautrin, F-54500 Vandoeuvre les Nancy, France; c.delattre@nancy.unicancer.fr; 8 Department of Geriatrics, Hôpital Européen Georges Pompidou, AP-HP, F-75015 Paris, France; 9 Department of Oncology, Dax Hospital, F-40100 Dax, France; daubaj@ch-dax.fr; 10 Department of Geriatrics, Centre Hospitalier Lyon Sud, Hospices Civils de Lyon, F-69310 Pierre-Bénite, France; gilles.albrand@chu-lyon.fr; 11 Department of Medical Oncology, Institut Curie, F-92210 Saint-Cloud, France; michael.bringuier@curie.fr (M.B.); etienne.brain@curie.fr (E.B.); 12 Department of Geriatrics, Bordeaux University Hospital, F-33000 Bordeaux, France; muriel.rainfray@chu-bordeaux.fr; 13 Epicene Team, Bordeaux Population Health Research Center, University Bordeaux, INSERM, UMR 1219, F-33000 Bordeaux, France; s.mathoulin@bordeaux.unicancer.fr (S.M.-P.); c.bellera@bordeaux.unicancer.fr (C.B.); 14 Clinical and Epidemiological Research Unit, Comprehensive Cancer Center, INSERM, CIC1401, Institut Bergonié, F-33000 Bordeaux, France

**Keywords:** cancer, older patients, mortality, biomarkers

## Abstract

**Simple Summary:**

The prognostic assessment of older cancer patients is complicated by their heterogeneity. We aimed to assess the prognostic value of routinely measured inflammatory biomarkers. We performed a pooled analysis of prospective multicenter cohorts of cancer patients aged ≥70. We measured CRP and albumin, and calculated Glasgow Prognostic Score (GPS) and CRP/albumin ratio. The GPS has three levels (0 = CRP ≤ 10 mg/L, albumin ≥ 35 g/L, i.e., normal values; 1 = one abnormal value; 2 = two abnormal values). Overall, 1800 patients were analyzed (mean age: 79 ± 6; males: 62%; metastases: 38%). The GPS and CRP/albumin ratio were independently associated with mortality. The discriminative power of the baseline clinical model was increased by adding GPS and CRP/albumin ratio. Routine inflammatory biomarkers add prognostic value to clinical factors in older cancer patients.

**Abstract:**

Background: The prognostic assessment of older cancer patients is complicated by their heterogeneity. We aimed to assess the prognostic value of routine inflammatory biomarkers. Methods: A pooled analysis of prospective multicenter cohorts of cancer patients aged ≥70 was performed. We measured CRP and albumin, and calculated Glasgow Prognostic Score (GPS) and CRP/albumin ratio. The GPS has three levels (0 = CRP ≤ 10 mg/L, albumin ≥ 35 g/L, i.e., normal values; 1 = one abnormal value; 2 = two abnormal values). One-year mortality was assessed using Cox models. Discriminative power was assessed using Harrell’s C index (C) and net reclassification improvement (NRI). Results: Overall, 1800 patients were analyzed (mean age: 79 ± 6; males: 62%; metastases: 38%). The GPS and CRP/albumin ratio were independently associated with mortality in patients not at risk of frailty (hazard ratio [95% confidence interval] = 4.48 [2.03–9.89] for GPS1, 11.64 [4.54–29.81] for GPS2, and 7.15 [3.22–15.90] for CRP/albumin ratio > 0.215) and in patients at risk of frailty (2.45 [1.79–3.34] for GPS1, 3.97 [2.93–5.37] for GPS2, and 2.81 [2.17–3.65] for CRP/albumin ratio > 0.215). The discriminative power of the baseline clinical model (C = 0.82 [0.80–0.83]) was increased by adding GPS (C = 0.84 [0.82–0.85]; NRI events (NRI+) = 10% [2–16]) and CRP/albumin ratio (C = 0.83 [0.82–0.85]; NRI+ = 14% [2–17]). Conclusions: Routine inflammatory biomarkers add prognostic value to clinical factors in older cancer patients.

## 1. Introduction

Individual prognostic assessments are crucial for treatment decisions in oncology. However, several studies have suggested that physicians are overly optimistic when predicting the survival of patients with terminal cancer [1]. Sixty percent of new cases of cancer occur in people aged 65 or over, and 30% occur in people aged 75 or over (World Health Organization 2014). Heterogeneity within this population, in terms of comorbidities and frailty profiles, implies that prognostic assessment is particularly challenging in older patients with cancer [2]. Moreover, older patients are underrepresented in clinical trials, resulting in a lack of knowledge about the risk-benefit ratio for cancer treatments in this age group [3].

Many studies have shown that assessment of geriatric parameters and use of frailty screening tools (such as the G8, the modified G8, and the Vulnerable Elders Survey-13) have prognostic value [4,5,6,7,8,9]. In particular, poor nutritional status, reduced mobility, poor functional status, and the presence of comorbidities are consistently and independently associated with a poor prognosis in older patients with cancer [4,10,11,12]. Several geriatric-oncologic scores have been developed for the prognostic assessment of this population [13,14,15].

These latter scores are based on clinical factors, such as age, sex, tumor site, functional status, comorbidity, cognitive status, affective status, or nutritional status, and none included biomarkers. Albumin and C-reactive protein (CRP) are both inflammatory markers, and albumin is also a nutritional marker. CRP is an acute-phase reaction protein, and its production is stimulated by interleukin-6, a cytokine released during the systemic inflammatory response to the tumor. [16] The increase in CRP is associated with an induction of inflammatory cytokines and increased activation of the complement and macrophage function, and this latter is closely associated with revascularization [17]. Albumin is both an inflammatory and a nutritional marker. Decreased albumin is associated with decreased body cell mass [18]; and hypoalbuminemia is part of the definition of cachexia, whose major cause is cytokine excess [19]. Thus, the decrease of albumin levels is considered to be part of the inflammatory response; indeed, interleukin-6 can adjust the synthesis of albumin by hepatocytes and contribute to lower albumin levels [16]. Inflammation in the tumor’s micro-environment leads to neovascularization, proliferation, angiogenesis, and metastasis [20,21]. Furthermore, inflammation is associated with progressive nutritional decline [19]. Moreover, several researchers have suggested that indices that combine CRP and albumin levels (such as the Glasgow Prognostic Score (GPS), the modified Glasgow Prognostic Score (mGPS), and the CRP/albumin ratio) may have more prognostic value than either marker alone [22,23,24]. Indeed, there is now good evidence that CRP and albumin alone and these indices have prognostic value in adult cancer populations [17,25]. We hypothesized that the readily available, routinely measured biomarkers CRP and albumin may have prognostic value in older cancer patients. Hence, we determined the predictive value of CRP and albumin (alone or combined) in older cancer patients and determined whether these biomarkers added value to a clinical prognostic model.

## 2. Methods

### 2.1. Study Design and Population

We performed a pooled analysis of three prospective multicenter cohort studies: the Elderly Cancer Patients (ELCAPA) cohort, the PHRC Aquitaine cohort, and the ONCODAGE cohort. All three studies comprise patients aged 70 or over with a diagnosed solid or hematologic cancer.

The ELCAPA cohort study (NCT02884375) has been enrolling patients diagnosed with cancer in hospitals in the Paris area of France since January 2007. The patients have been referred for a geriatric assessment by oncologists, radiation therapists, or surgeons before any first-line treatment or between any two steps of a previously scheduled first-line treatment sequence [26].

The PHRC 2003 Aquitaine cohort (NCT00210249) recruited patients with an indication of first-line chemotherapy and no previous treatment between September 2002 and September 2005 at 12 centers in the Aquitaine region of France. The patients had various types of cancer (colon, pancreas, stomach, ovary, bladder, prostate, lung cancer, non-Hodgkin’s lymphoma, or cancer of unknown primary origin), with the exception of breast cancer. The CGA was performed after an initial oncology consultation [27].

The ONCODAGE study (NCT00963911) included patients consulting before any first-line treatment or between any two steps of a previously scheduled first-line treatment sequence for various types of cancer (colon, lung, upper aerodigestive tract/head and neck, breast, prostate, and non-Hodgkin’s lymphoma). They were included between August 2008 and March 2010 by healthcare facilities throughout France (and notably 15 regional geriatric oncology coordination units) [8].

We performed a pooled analysis of patients included in any of the three studies. Additional eligibility criteria for this pooled analysis included: availability of data on plasma albumin and plasma CRP as well as one-year vital status. Patients with hematologic malignancies (except lymphoma) were excluded from the analysis. Patients between any two steps of a previously scheduled first-line treatment sequence could be included as it was mostly surgery.

### 2.2. Ethical Considerations

Each cohort study was carried out in accordance with the Declaration of Helsinki, good clinical practice, and French legislation on clinical research. All studies had been approved by an institutional review board, and all patients had provided their informed consent prior to inclusion.

### 2.3. Data Recorded

At baseline, we prospectively recorded sociodemographic data (age, sex, living alone or not, etc.), primary tumor site, metastatic status, and Eastern Cooperative Oncology Group performance status (ECOG-PS). The latter was dichotomized as grades 0–1 (normal functional status) and grades 2–4 (abnormal functional status). Metastatic status was classified as M0 (no distant metastases), M1 (distant metastases), Mx (metastasis status unknown), or NA (not applicable). Primary tumor sites were categorized as follows: colorectal, breast, prostate, lung and bronchial, head and neck, other digestive tract sites (esophagus, stomach, biliary tract, hepatocellular carcinoma), other urinary tract sites (kidney, bladder, urethra, etc.), hematologic, and other sites (skin, central nervous system, sarcoma, thyroid, unknown primary site, synchronous sites, uterus, ovary, small intestine, peritoneal, penile, or germ cell tumor).

The risk of frailty was assessed using the G8 score which ranges from 0 to 17, and a score of 14 or less corresponds to a risk of frailty (i.e., a requirement for geriatric assessment) (Supplementary Methods) [27]. The modified G8 was also recorded. It ranges from 0 to 35, and a score of 6 or more corresponds to a risk of frailty [28] (Supplementary Methods).

We used the collected values on plasma levels of CRP (abnormal if >10 mg) and albumin (abnormal if <35 g/L) to calculate the GPS and the mGPS. The GPS has three levels (level 0: CRP ≤ 10 mg/L and albumin ≥ 35 g/L, i.e., normal values for both biomarkers; level 1: CRP ≤ 10 mg/L and albumin < 35 g/L, or CRP > 10 mg/L and albumin ≥ 35 g/L, i.e., one abnormal value; level 2: CRP > 10 mg/L and albumin < 35 g/L, i.e., two abnormal values). The mGPS also has three levels but weights the CRP component more heavily (level 0: CRP ≤ 10 mg/L and albumin ≥ 35 g/L or CRP ≤ 10 mg/L and albumin < 35 g/L; level 1: CRP > 10 mg/L whatever albumin value). The CRP/albumin ratio was also calculated.

A geriatric assessment was also performed. Functional status was evaluated using the Activities of Daily Living (ADL) scale (dependence if ≤5) and the Instrumental Activities of Daily Living (IADL) scale (dependence if <8). Mobility was assessed using the timed get-up-and-go test; impaired mobility was defined as a score > 20 s. Malnutrition was defined as a BMI < 21 kg/m^2^, a Mini Nutritional Assessment (MNA) < 17 out of 30, plasma albumin < 35 g/L, or recent weight loss (defined as loss of more than 3 kg in the last three months according to MNA criteria). Mood was evaluated on the four-item or 15-item Geriatric Depression Scale (GDS-4 and -15, respectively). Patients were classified as being at risk of depression if the GDS-4 score was ≥1 out of 4 or the GDS-15 score was ≥6 out of 15. Cognitive function was assessed using the Mini Mental State Examination (MMSE), and patients were classified as being at risk of dementia if the MMSE score was below 24 out of 30. Comorbidities were noted on the Cumulative Illness Rating Scale (CIRS-G). Renal function was also recorded by calculating the estimated glomerular filtration rate (eGFR) according to the Cockcroft-Gault formula.

## 3. Endpoints

The primary endpoint was one-year mortality, defined as the time from baseline to death or to the last follow-up. Patients were censored at one-year follow-up when alive at this time point or at last follow-up when lost to follow-up before one year. Vital status at one year was ascertained using medical records or public records office archives.

### Statistical Analyses

Categorical variables were quoted as the number (percentage), and quantitative variables were quoted as the mean ± standard deviation (SD) or the median [interquartile range (IQR)], as appropriate.

Survival in the total population and in subgroups defined by biomarker levels was evaluated using the Kaplan–Meier method. The results were expressed as the survival rate [95% confidence interval (CI 95%)].

Univariate and multivariable Cox proportional hazards models were used to analyze the biomarkers’ prognostic value. An initial model was built with clinical variables known to be predictors of death in patients with cancer (i.e., age, sex, tumor site, metastatic status, performance status, and the G8 frailty screening score) [4,6,10]. We then built five models with biomarkers and the derived scores, each one added to the clinical variables in five separate models. The biomarker was treated as the main exposure variable. Biomarkers were considered as binary variables by applying the above-mentioned cut-offs, which are those used in clinical practice. The best cut-off for the CRP/albumin ratio (i.e., that which maximized both sensitivity and specificity for mortality) was determined using the Youden index. It is an index corresponding to sensitivity + specificity − 1, which is commonly used to define the best cut-point of a continuous variable since it maximizes the number of correctly classified subjects [29,30].

We tested the interactions between the tumor site and metastatic status in the baseline model, and between respectively each biomarker, i.e., our main variable of interest, and clinical variables, using likelihood ratios tests in the final models. The results were expressed as the hazard ratio (HR) [95% CI]. The proportional hazard assumption was checked by plotting the Schoenfeld residuals. Goodness-of-fit was assessed by plotting the Cox-Snell residuals.

Discriminant ability was assessed using Harrell’s C index and the net reclassification improvement (NRI). The NRI corresponds to the proportions of patients correctly reclassified with an event (“NRI+” or “NRI event”) and without an event (“NRI−” or “NRI non-event”) after a variable has been added to a baseline model. Hereafter, NRI+ for a given model with a biomarker refers to the proportion of patients who died but had been classified as not being at risk of one-year mortality in the baseline clinical model and were correctly reclassified as being at risk of one-year mortality after the biomarker had been added. Similarly, NRI− was defined as the proportion of patients who did not die and were classified as being at risk of one-year mortality in the baseline model and were correctly reclassified as not being at risk of one-year mortality after the biomarker had been added [31,32,33].

Subgroup analyses were performed as a function of metastatic status. A sensitivity analysis was performed by using the modified G8 score, with the same methodology as for the primary analysis.

All significance tests were two-tailed, and the threshold for statistical significance level was set to 5%. All analyses were performed with Stata software (version 14.1, StataCorp, College Station, TX, USA) and R software (R Core Team. R: A language and environment for statistical computing. R Foundation for Statistical Computing, Vienna, Austria, version 3.4.4) [34].

## 4. Results

**Figure 1:**
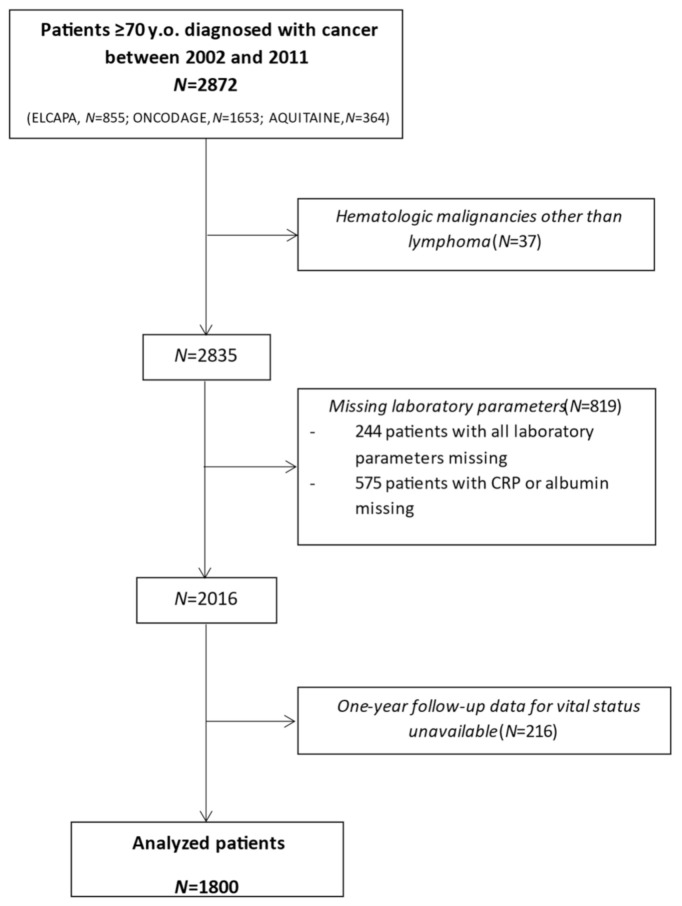
Study flowchart.

A total of 1800 patients were analyzed (ELCAPA: *n* = 543, PHRC Aquitaine: *n* = 253, ONCODAGE: *n* = 1004) (Figure 1). The mean ± SD age was 79 ± 6, there was male predominance (62%), and 29% of the patients had metastatic cancer. The most frequent primary tumor locations were the breast (35%) and colon/rectum (18%). According to the G8, 71% of patients were at risk of frailty. The characteristics of the study population are summarized in Table 1.

The median [IQR] follow-up time was 15.6 months [12.1–40.7]. The one-year mortality rate was 28.9% [95% CI: 26.9; 31.1].

In a univariate analysis, age, sex, tumor site, metastatic status, altered ECOG-PS (≥2), and altered G8 (≤14) were associated with one-year mortality. With regard to the biomarkers, CRP, albumin, the GPS score, the mGPS score and the CRP/albumin ratio (>0.215) were significantly associated with one-year mortality (crude HR [95% CI] = 5.14 [4.24–6.24] for CRP > 10 mg/L, 4.92 [4.11–5.90] for albumin < 35 g/L, 4.54 [3.49–5.91] for GPS 1, 10.82 [8.45–13.85] for GPS 2, 3.00 [2.33–3.86] for mGPS 1, 7.26 [5.92–8.92] for mGPS 2, and 6.29 [5.06–7.80] for the CRP/albumin ratio) (Table 2).

We observed a significant interaction between tumor site and metastatic status (*p* for interaction = 0.02) and between the G8 score and the biomarker levels (*p* = Table S1). Accordingly, a variable that combined tumor site and metastatic status was built, and each model included a term for the interaction between G8 on one hand and GPS, mGPS, CRP, albumin, and CRP/albumin ratio on the other.

**Figure 2:**
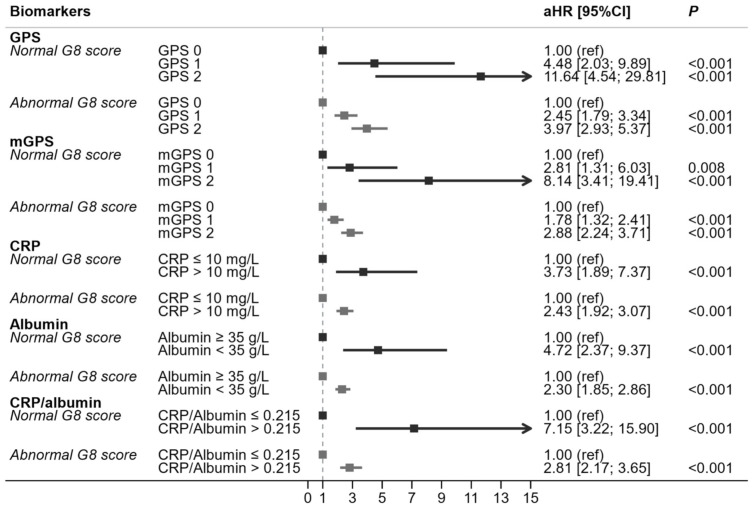
Multivariable analysis of GPS, mGPS, CRP, albumin and CRP/albumin ratio with regard to one-year mortality (*n* = 1604).

**Figure 3:**
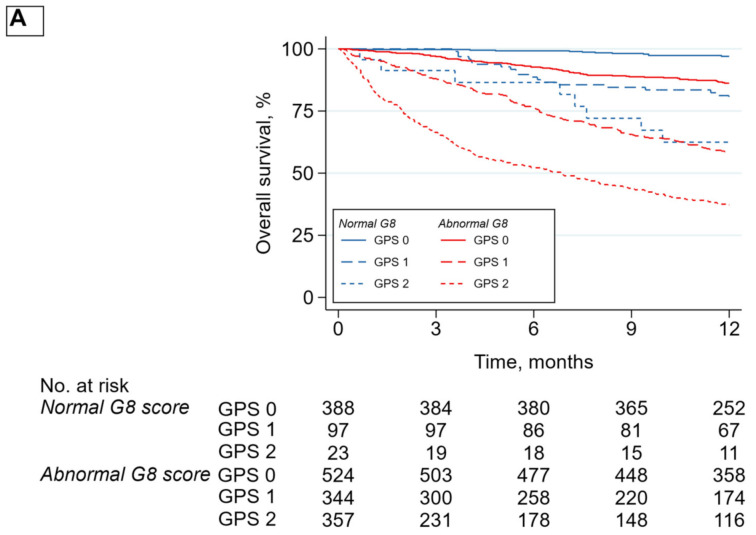

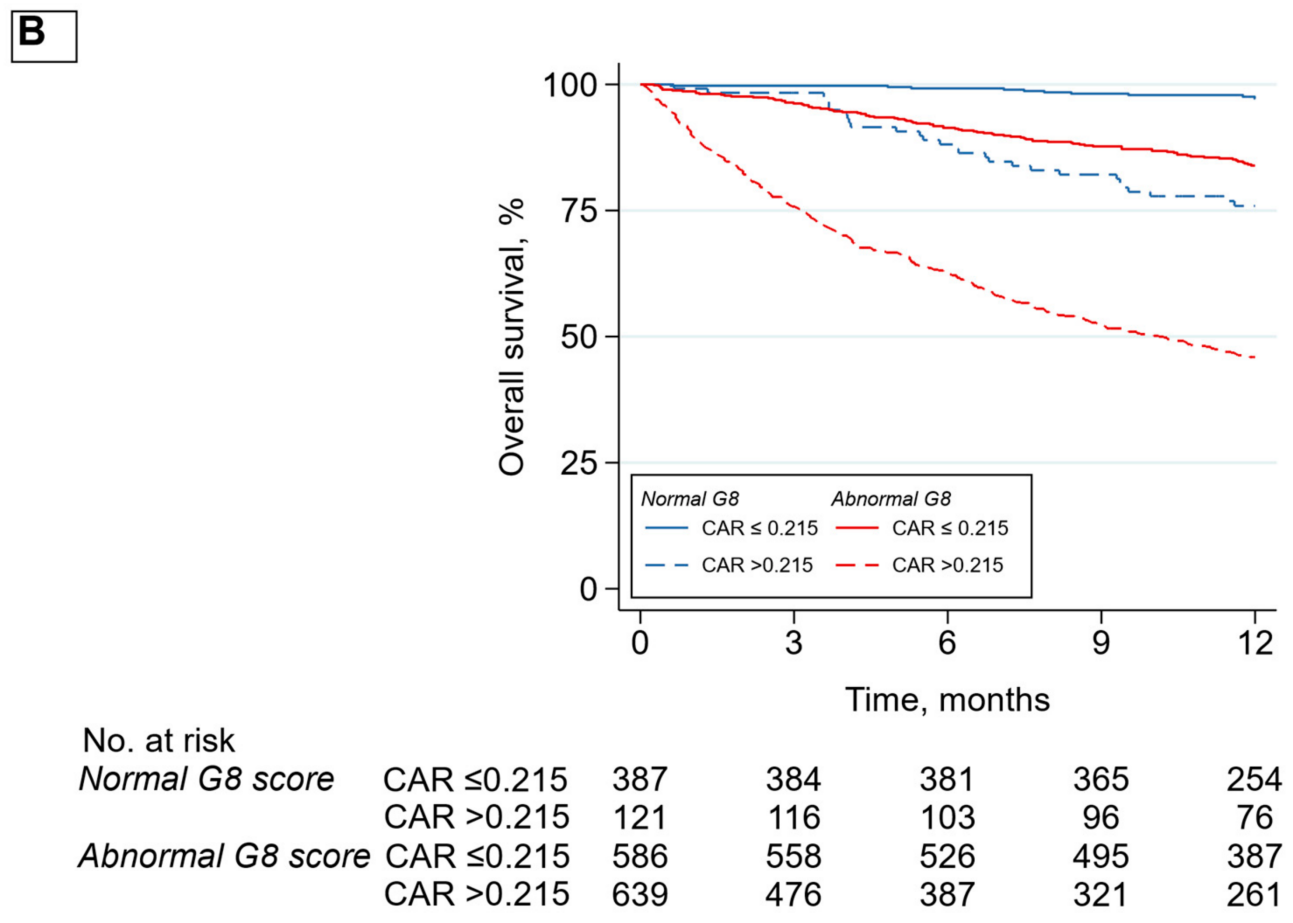
Overall survival according to GPS (**A**) and CRP/albumin ratio (**B**), stratified by G8 score (*n* = 1800). GPS: Glasgow Prognostic Score; CAR: C-reactive protein/albumin ratio.

In multivariable analyses, plasma CRP, plasma albumin, the GPS score, the mGPS score, and the CRP/albumin ratio were respectively and independently associated with mortality. The association between each inflammatory biomarker and mortality was stronger in patients not at risk of frailty (according to the G8) than in patients at risk of frailty. (Figure 2 and Figure 3).

Adjusted hazard ratios (aHR) and *p*-values correspond to multivariable analysis models adjusted for age, sex, tumor site, metastatic status, ECOG-PS (Eastern Cooperative Oncology Group-Performance Status), G8 frailty screening score, with a term for the interaction between G8 and the biomarker (one model per biomarker, with each biomarker added singly to the baseline clinical model).

GPS: Glasgow Prognostic Score; mGPS: modified GPS; CRP: C-reactive protein; aHR: adjusted hazard ratio; CI: confidence interval.

The discriminant power of the baseline clinical model was very high (Harrell’s C = 0.82 [0.80; 0.83]). The addition of each biomarker or related score to the clinical model increased the discriminant power with greatest increases for the GPS, the CRP/albumin ratio and the mGPS (Harrell’s C = 0.84 [0.82; 0.85], 0.83 [0.82; 0.85] and 0.83 [0.82; 0.85], respectively; NRI+: 10% [2; 16], 8% [−3; 14] and 14% [2; 17], respectively) (Table 3). For all the biomarkers, the increase in discriminant power mainly concerned NRI+, i.e., patients who had died within one year and were correctly reclassified as being at risk of one-year mortality (Table 3). The sensitivity analysis using the modified G8 gave similar results (Supplementary Tables S2 and S3).

In the subgroup of patients with metastatic cancer, the addition of biomarkers improved Harrell’s C index and (albeit to a lesser extent) the NRI (Supplementary Tables S4 and S5). The limited number of patients with non-metastatic cancer prevented an analysis of this subgroup (137 events, 16 parameters to estimate).

## 5. Discussion

Our present results showed that routinely measured inflammatory biomarkers (albumin and CRP) and combinations thereof (the GPS, the mGPS, and the CRP/albumin ratio) were respectively and independently associated with one-year mortality in older cancer patients. Hence, these biomarkers add prognostic value to conventional demographic, oncological, and frailty risk factors. Each of the three composite markers (the GPS, the CRP/albumin ratio, and the mGPS) had better additional prognostic and discriminative value than either of the plasma biomarkers alone. The associations between inflammatory biomarkers and mortality were stronger in fit patients than in patients at risk of frailty.

Our present results are in line with the literature data on adults with cancer. Indeed, CRP and albumin are known to have prognostic value in middle-aged adults [17,35]. Guner et al. showed that albumin had a greater area under the ROC curve (calculated using both sensitivity and specificity), and thus a greater discriminant power than neutrophil, lymphocyte, and platelet counts and combinations thereof [35].

Regarding scores in which CRP and albumin are combined, two recent meta-analyses in adults by Dolan et al. showed that an elevated GPS/mGPS was predictive of mortality in patients with operable cancers and in those with inoperable cancer [36,37]. Our results for the CRP/albumin ratio were also consistent with the meta-analysis by Xu et al., according to which a high CRP/albumin ratio was predictive of mortality in adults with different types of cancer). The cut-off values ranged from 0.028 to 0.54 [24].

The Glasgow Prognostic Score or modified Glasgow Prognostic Score have been shown to be predictive of mortality in other diseases than cancer, such as acute pancreatitis [38], heart failure [39,40], acute pulmonary embolism [41] or acute exacerbation of chronic obstructive pulmonary disease [42]. The CRP albumin ratio has also been shown predictive of mortality in patients without cancer for example in patients having systemic lupus with serious community-acquired infection [43] or in critically ill patients [44].

To the best of our knowledge, only a few studies have explored the added value of GPS, mGPS, or CRP/albumin ratio in older cancer patients. With regard to mGPS, Hirashima et al., who focused on gastric cancer in a subgroup of patients aged 75 or over, found that this score was significantly associated with all-cause mortality [45]. Regarding GPS, Miyazaki et al. and Ohki et al.’s showed that the GPS score was a prognostic factor for 5-year all-cause mortality in studies of over-80 patients with respectively clinical stage I non-small-cell lung cancer and colorectal cancer [46,47]. Recently, Baitar et al. showed that CRP, albumin, GPS, and mGPS added value to baseline clinical factors in 328 patients with various tumor sites. However, their baseline clinical model did not include sex, performance status, or the frailty risk [48]. Regarding the CRP/albumin ratio, Miyazaki et al. reported that it had prognostic value (for predicting death) in older patients with resectable non-small-cell lung cancer [49], as we reported for older patients with metastatic non-small-cell lung cancer [50]. Furthermore, in a random survival forest analysis of clinical factors and laboratory variables in older patients with various types of cancer, we showed that the CRP/albumin ratio made the largest contribution to the prediction of one-year mortality [51].

The associations between inflammatory biomarkers and mortality found to be stronger in fit patients than in patients at risk of frailty could be explained by the fact that in patients at risk of frailty, the association between biomarkers and mortality could be mediated by other factors such as items of the G8 such as weight loss, mobility skills that are more altered in the abnormal G8 group than in the normal G8 group, in which this mediation phenomenon might not occur.

Our study had a number of strengths. It was a pooled analysis of three prospective cohort studies with similar designs. The fact that plasma levels of CRP and albumin are routinely measured in cancer older patients underlines the clinical applicability of our present results.

Our study also had some limitations. Firstly, the biomarker levels were not measured centrally. Secondly, the low number of events in some subgroups prevented us from studying the biomarkers’ prognostic values for the different tumor sites. Thirdly, we cannot fully rule out the presence of residual confounding factors, such as parameters in the geriatric assessment. A comparative analysis of the value of these inflammation-based biomarkers vs. geriatric parameters would be of interest.

In line with the reports by Retornaz et al. [52] and our team [51], our present work shows that biomarkers can be included in prognostic scores for older cancer patients. Our results suggest that these routinely measured inflammatory biomarkers can be used in the clinic to assess the prognosis of older cancer patients—including fit patients with a normal G8 score, who might not be referred to a geriatrician. Future studies, to explore the prognostic value of these biomarkers, measured at different time points, would be useful.

## 6. Conclusions

In conclusion, we found that two routinely measured inflammatory biomarkers (albumin and CRP) and the scores calculated therefrom (the GPS, the mGPS, and the CRP/albumin ratio) add prognostic value to conventional demographic, oncologic or frailty risk factors when considering older cancer patients. The consideration of these biomarkers could facilitate clinical decision-making with regard to older cancer patients.

## Figures and Tables

**Table 1:** Baseline characteristics of the study population.

	No. (%) Total (*n* = 1800)
**Age**, mean ± SD	79 ± 6
**Female sex**	690 (38)
**Tumor site**	
Colon/rectum	318 (18)
Pancreas	52 (3)
Other digestive tract malignancies ^a^	74 (4)
Breast	629 (35)
Prostate	173 (10)
Other urinary tract malignancies ^b^	90 (5)
Lung and bronchial	141 (8)
Head and neck	62 (3)
Lymphoma	205 (11)
Other cancers ^c^	56 (3)
**Metastatic status** (*n* = 1735) ^d^	
M0	864 (50)
M1	502 (29)
Mx	164 (9)
NA	205 (12)
**Social characteristics**	
Living alone (*n* = 1793)	719 (40)
**Functional status**	
ADL score ≤ 5 out of 6 (*n* = 1794)	601 (34)
IADL score < 8 out of 8 (*n* = 1690)	951 (56)
ECOG-PS ≥ 2 out of 4 (*n* = 1729)	559 (32)
TGUG > 20 s (*n* = 1514)	213 (14)
**Malnutrition**^e^ (*n* = 1774)	1004 (57)
**G8** score ≤ 14 (*n* = 1733)	1225 (71)
**Cognitive and emotional status**	
MMSE < 24 out of 30 (*n* = 1687)	371 (22)
GDS-15 ≥ 6 out of 15 or GDS4 ≥ 1 out of 4 (*n* = 1664)	580 (35)
**Comorbidity**	
CIRS-G, median [IQR] (*n* = 1741)	9 [6–13]
CIRS-G grade 3 or 4 (*n* = 1747)	932 (53)
Renal insufficiency (Cockcroft-Gault, mL/min) (*n* = 1755)	
absent (clearance ≥ 60)	831 (47)
moderate (30 ≤ clearance < 60)	825 (47)
severe (clearance < 30)	99 (6)
**Inflammatory biomarkers**	
CRP > 10 mg/L (*n* = 1800)	716 (39.78)
CRP, median [IQR] (*n* = 1800)	6 [2.70–27.25]
Albumin < 35 g/L (*n* = 1800)	586 (33)
Albumin, median [IQR] (*n* = 1800)	38.0 [33.00–42.00]
Prealbumin < 140 mg/L, (*n* = 1497)	262 (18)
GPS (*n* = 1800)	
GPS = 0	921 (51)
GPS = 1	456 (25)
GPS = 2	423 (24)
mGPS (*n* = 1800)	
mGPS = 0	1084 (60)
mGPS = 1	293 (16)
mGPS = 2	423 (24)
CRP/albumin ratio > 0.215 (*n* = 1800)	816 (45)
CRP/albumin ratio, median [IQR] (*n* = 1800)	0.17 [0.07–0.80]

Abbreviations: ELCAPA: elderly cancer patients; ADL: activities of daily living; IADL: instrumental activities of daily living; ECOG-PS: Eastern Cooperative Oncology Group performance status; TGUG: timed get-up-and-go test; MMSE: Mini-Mental State Examination; GDS: Geriatric Depression Scale; CIRS -G: Cumulative Illness Rating Scale; BMI: body mass index; MNA: Mini-Nutritional Assessment; CRP: C-reactive protein; GPS: Glasgow Prognostic Score; mGPS: modified Glasgow Prognostic Score; IQR: interquartile range; SD: standard deviation. ^a^ Other digestive tract malignancies: esophagus, stomach, biliary tract, hepatocellular carcinoma. ^b^ Other urinary tract malignancies: kidney, bladder, urinary tract. ^c^ Other cancer sites: skin; central nervous system, sarcoma, thyroid, unknown primary, synchronous, uterus, ovary, small intestine, peritoneal, penile, germ cell tumor. ^d^ Metastatic status: data available for *n* = 1530 among 1595 (lymphoma excluded); patients with lymphoma (*n* = 205) are considered NA with regard to metastatic status. ^e^ Malnutrition defined as any of BMI < 21 kg/m^2^, MNA < 17/30, albumin < 35 g/L, and recent weight loss (defined as loss of more than 3 kg in the last three months according to MNA criteria).

**Table 2:** Univariate analysis of clinical and laboratory variables associated with one-year mortality (*n* = 1800).

	One-Year Survivors (*n*, %) *n* = 1294	Deceased at One Year (*n*, %) *n* = 506	Unadjusted Hazard Ratio [95% CI]	*p*
**Age**, mean ± SD	78 ± 5	80 ± 6	1.05 [1.04–1.07]	<0.001
**Female sex**	465 (36)	225 (44)	1.36 [1.14–1.62]	0.001
**Tumor site**				
Colorectal	222 (17)	96 (19)	1.00 (ref)	<0.001
Pancreas	11 (1)	41 (8)	4.73 [3.28–6.83]	
Other digestive tract malignancies ^a^	35 (3)	39 (8)	2.03 [1.40–2.95]	
Breast	572 (44)	57 (11)	0.26 [0.19–0.37]	
Prostate	136 (11)	37 (7)	0.67 [0.46–0.99]	
Other urinary tract malignancies ^b^	44 (3)	46 (9)	1.99 [1.40–2.82]	
Lung and bronchial	64 (5)	77 (15)	2.23 [1.65–3.01]	
Head and neck	34 (3)	28 (6)	1.62 [1.06–2.46]	
Hematologic	160 (12)	45 (9)	0.69 [0.48–0.98]	
Other cancers ^c^	16 (1)	40 (8)	3.41 [2.35–4.93]	
**Metastatic status** (*n* = 1735)				
M0	714 (57)	150 (31)	1.00 (ref)	<0.001
M1	234 (19)	268 (55)	4.12 [3.37–5.04]	
Mx	142 (11)	22 (5)	0.76 [0.49–1.20]	
NA	160 (13)	45 (9)	1.31 [0.94–1.83]	
**ECOG-PS** ≥ 2 out of 4	258 (21)	301 (62)	4.84 [4.03–5.82]	<0.001
**G8** score ≤ 14	797 (63)	428 (92)	5.62 [4.03–7.84]	<0.001
**Biomarkers**				
CRP > 10 mg/L	354 (27)	362 (72)	5.14 [4.24–6.24]	<0.001
Albumin < 35 g/L	268 (21)	318 (63)	4.92 [4.11–5.90]	<0.001
GPS				
GPS = 0	837 (65)	84 (17)	1.00 (ref)	<0.001
GPS = 1	292 (22)	164 (32)	4.54 [3.49–5.91]	
GPS = 2	165 (13)	258 (51)	10.82 [8.45–13.85]	
mGPS				
mGPS = 0	940 (73)	144 (28)	1.00 (ref)	<0.001
mGPS = 1	189 (14)	104 (21)	3.00 [2.33–3.86]	
mGPS = 2	165 (13)	258 (51)	7.26 [5.92–8.92]	
CRP/albumin ratio > 0.215	414 (32)	402 (79)	6.29 [5.06–7.80]	<0.001

Abbreviations: ELCAPA: elderly cancer patients; ECOG-PS: Eastern Cooperative Oncology Group-Performance Status; CRP: C-reactive protein; GPS: Glasgow Prognostic Score; mGPS: modified Glasgow Prognostic Score; IQR: interquartile range; SD: standard deviation; CI: confidence interval. ^a^ Other digestive tract malignancies: esophagus, stomach, biliary tract, hepatocellular carcinoma. ^b^ Other urinary tract malignancies, kidney, bladder, urinary tract. ^c^ Other cancer sites: skin, central nervous system, sarcoma, thyroid, unknown primary, synchronous, uterus, ovary, small intestine, peritoneal, penile, germ cell tumor.

**Table 3:** Discriminant power of models with GPS, mGPS, CRP, albumin and CRP/albumin ratio with regard to one-year mortality in the overall population (*n* = 1604 for all models).

Model	Harrell’s C [95% CI]	NRI+ [95% CI]	NRI− [95% CI]
Baseline model: age, sex, tumor site, metastatic status, ECOG-PS, G8	0.82 [0.80; 0.83]	-	-
Baseline model + **GPS**	0.84 [0.82; 0.85]	0.10 [0.02; 0.16]	0.01 [−0.02; 0.09]
Baseline model + **mGPS**	0.83 [0.82; 0.85]	0.08 [−0.03; 0.14]	0.01 [−0.03; 0.09]
Baseline model + **CRP**	0.83 [0.82; 0.85]	0.11 [−0.01; 0.15]	−0.01 [−0.05; 0.09]
Baseline model + **albumin**	0.83 [0.81; 0.85]	0.07 [−0.04; 0.13]	0.00 [−0.04; 0.09]
Baseline model + **CRP/albumin ratio**	0.83 [0.82; 0.85]	0.14 [0.02; 0.17]	−0.01 [−0.05; 0.08]

ECOG-PS: Eastern Cooperative Oncology Group performance status; CRP: C-reactive protein; GPS: Glasgow Prognostic Score; mGPS: modified Glasgow Prognostic Score; NRI: net reclassification improvement; CI: confidence interval.

## Data Availability

The datasets analyzed during the current study are not publicly available because they are the property of Assistance Publique Hôpitaux de Paris and Institut Bergonié.

## Supplementary Materials

The following are available online at https://www.mdpi.com/article/10.3390/cancers13246154/s1. Table S1: Multivariable analysis of GPS, mGPS, CRP, albumin and the CRP/albumin ratio with regard to one-year mortality: a sensitivity analysis with the modified G8 score (*n* = 1334 for all models); Table S2: Discriminant power of models with GPS, mGPS, CRP, albumin and CRP/albumin ratio: a sensitivity analysis with the modified G8 screening score (*n* = 1334); Table S3: Multivariable analysis of GPS, mGPS, CRP, albumin and CRP/albumin ratio with regard to one-year mortality in the subgroup of patients with metastatic cancer (*n* = 666); Table S4: Discriminant power of models with GPS, mGPS, CRP, albumin and CRP/albumin ratio with regard to one-year mortality in metastatic cancer subgroup (*n* = 666); Table S5: Discriminant power of models with GPS, mGPS, CRP, albumin and CRP/albumin ratio with regard to one-year mortality in metastatic cancer subgroup (*n* = 666).
