# Supplementary material for: Prognostic Value of Routinely Measured Inflammatory Biomarkers in Older Cancer Patients: Pooled Analysis of Three Cohorts
Source: Cancers (Basel). 2021 Dec 7;13(24):6154. doi: 10.3390/cancers13246154 (PMC8699299; doi:10.3390/cancers13246154)
Supplement: Supplementary file 1 [file cancers-13-06154-s001.zip › cancers-1461041-supplementary.pdf]

Supplementary

Prognostic value of routinely measured inflammatory biomarkers in older cancer patients: pooled analysis of three cohorts.

Supplementary Methods

The risk of frailty was assessed using the G8 score, which comprises eight items [24]: food intake (A), weight loss (B), mobility/motor skills (C), neuropsychological problems (D), body mass index (BMI) (E), number of medications (F), self-perception of health (G), and age (H). Items A, C, D, G and H were graded as 0 (least favorable state), 1, or 2 (most favorable state); items B and E were graded as 0, 1, 2, or 3 (where 3 is the most favorable state), and item F was graded as either 0 or 1 (where 1 is the most favorable state). The total G8 score ranges from 0 to 17, and a score of 14 or less corresponds to a risk of frailty (i.e. a requirement for geriatric assessment).

The modified G8 was also recorded; it contains six items [25]: weight loss, graded as 10, 2 and 0 (most favorable state), neuropsychological status, graded as 3 or 0 (most favorable state), number of medications, graded as 2 or 0 (most favorable state), self-perception of health, graded as 3 or 0 (most favorable state), performance status, graded as 12, 4 or 0 (most favorable state), and a history of heart failure or coronary artery disease, graded as 5 or 0 (most favorable state). The total modified G8 score ranges from 0 to 35, and a score of 6 or more corresponds to a risk of frailty.

References

[27] Bellera, C.A.; Rainfray, M.; Mathoulin-Pelissier, S.; Mertens, C.; Delva, F.; Fonck, M.; Soubeyran, P.L. Screening older cancer patients: first evaluation of the G-8 geriatric screening tool. Annals of oncology : official journal of the European Society for Medical Oncology 2012, 23, 2166-2172, doi:10.1093/annonc/mdr587.
[28] Martinez-Tapia, C.; Canoui-Poitrine, F.; Bastuji-Garin, S.; Soubeyran, P.; Mathoulin-Pelissier, S.; Tournigand, C.; Paillaud, E.; Laurent, M.; Audureau, E. Optimizing the G8 Screening Tool for Older Patients With Cancer: Diagnostic Performance and Validation of a Six-Item Version. The oncologist 2016, 21, 188-195, doi:10.1634/theoncologist.2015-0326.

Table S1: Multivariable analysis of GPS, mGPS, CRP, albumin and the CRP/albumin ratio with regard to one-year mortality (n=1604 for all models)

Models	Adjusted hazard ratio [95%CI]	*P*
G8 score		
Normal	1	
Abnormal	2.90 [1.46; 5.78]	0.002
GPS		
GPS 0	1	
GPS 1	4.48 [2.03; 9.89]	<0.001
GPS 2	11.64 [4.54; 29.81]	<0.001
Abnormal G8 score*GPS 1	0.55 [0.24; 1.27]	0.16
Abnormal G8 score*GPS 2	0.34 [0.13; 0.91]	0.03
G8 score		
Normal	1	
Abnormal	2.61 [1.48; 4.60]	0.001
mGPS		
mGPS 0	1	
mGPS 1	2.81 [1.31; 6.03]	0.008
mGPS 2	8.14 [3.41; 19.41]	<0.001
Abnormal G8 score*mGPS 1	0.63 [0.28; 1.43]	0.27
Abnormal G8 score*mGPS 2	0.35 [0.14; 0.87]	0.02
G8 score		
Normal	1	
Abnormal	2.55 [1.44; 4.50]	0.001
CRP		
CRP ≤10 mg/L	1	
CRP >10 mg/L	3.73 [1.89; 7.37]	<0.001
Abnormal G8 score*CRP >10 mg/L	0.65 [0.32; 1.33]	0.24
G8 score		
Normal	1	
Abnormal	2.25 [1.42; 3.58]	0.001
Albumin		
Albumin ≥35 g/L	1	
Albumin <35 g/L	4.72 [2.37; 9.37]	<0.001
Abnormal G8 score*Albumin <35 g/L	0.49 [0.24; 0.99]	0.049
G8 score		
Normal	1	
Abnormal	3.94 [1.86; 8.32]	<0.001
CRP/albumin ratio		
CRP/albumin ratio ≤0.215	1	
CRP/albumin ratio >0.215	7.15 [3.22; 15.90]	<0.001
Abnormal G8 score*CRP/albumin ratio >0.215	0.39 [0.17; 0.90]	0.03

Hazard ratios and p-values correspond to multivariable analysis models adjusted for age, sex, tumor site, metastatic status, ECOG-PS, and G8 frailty screening score, with a term for the interaction between G8 and the biomarker (one model per biomarker, with each biomarker added singly to the baseline clinical model). Results are interpreted as follows: for example for GPS, Abnormal G8 score*GPS 1 and Abnormal G8 score*GPS 2 correspond to the interactions terms; HR for G8 score and for GPS correspond to stratum-specific HR, i.e. the HR for G8 score corresponds to the HR of the association between abnormal G8 score and one-year mortality in the stratum where GPS=0 (reference value of the variable

GPS); the HR for GPS1 and GPS2 correspond respectively to the asciation between GPS1 and one-year mortality and the association between GPS2 and one-year mortality in the stratum of patients with a normal G8 score (reference value of the variable GS score). And for example, to get the HR of the association between GPS2 and one-year mortality in the stratum of patients with an abnormal G8 score, we multiply the HR of the association between GPS2 and one-year mortality in the stratum of patients with a normal G8 score (i.e. 11.64) by the interaction term Abnormal G8 score*GPS 2 (i.e. 0.34), which gives an HR of 3.97 as presented in Figure 2.
GPS: Glasgow Prognostic Score; mGPS: modified Glasgow Prognostic Score; CRP: C-reactive protein; CI: confidence interval.

Table S2: Multivariable analysis of GPS, mGPS, CRP, albumin and the CRP/albumin ratio with regard to one-year mortality: a sensitivity analysis with the modified G8 score (n=1334 for all models)

Models	Adjusted hazard ratio [95%CI]	*P*
Normal modified G8 score		
GPS 0	1	
GPS 1	3.98 [1.48; 10.73]	0.006
GPS 2	7.42 [1.94; 28.39]	0.003
Abnormal modified G8 score		
GPS 0	1	
GPS 1	2.68 [1.87; 3.83]	<0.001
GPS 2	4.26 [3.00; 6.06]	<0.001
Normal modified G8 score		
mGPS 0	1	
mGPS 1	4.33 [1.59; 11.80]	0.004
mGPS 2	6.60 [1.76; 24.75]	0.005
Abnormal modified G8 score		
mGPS 0	1	
mGPS 1	1.77 [1.25; 2.49]	0.001
mGPS 2	2.86 [2.15; 3.80]	<0.001
Normal modified G8 score		
CRP ≤10 mg/L	1	
CRP >10 mg/L	4.65 [1.86; 11.61]	0.001
Abnormal modified G8 score		
CRP ≤10 mg/L	1	
CRP >10 mg/L	2.41 [1.85; 3.14]	<0.001
Normal modified G8 score		
Albumin ≥35 g/L	1	
Albumin <35 g/L	2.62 [0.86; 7.94]	0.09
Abnormal modified G8 score		
Albumin ≥35 g/L	1	
Albumin <35 g/L	2.41 [1.87; 3.11]	<0.001
Normal modified G8 score		
CRP/albumin ratio ≤0.215	1	
CRP/albumin ratio >0.215	7.78 [2.93; 20.67]	<0.001
Abnormal modified G8 score		
CRP/albumin ratio ≤0.215	1	
CRP/albumin ratio >0.215	2.98 [2.20; 4.03]	<0.001

Hazard ratios and p-values correspond to multivariable analysis models adjusted for age, sex, tumor site, metastatic status, ECOG-PS, and G8 frailty screening score, with a term for the interaction between G8 and the biomarker (one model per biomarker, with each biomarker added singly to the baseline clinical model).
GPS: Glasgow Prognostic Score; mGPS: modified Glasgow Prognostic Score; CRP: C-reactive protein; CI: confidence interval.

Table S3: Discriminant power of models with GPS, mGPS, CRP, albumin and CRP/albumin ratio: a sensitivity analysis with the modified G8 screening score (n=1334)

**Model**	**Harrell's C [95%CI]**	**NRI+ [95%CI]**	**NRI- [95%CI]**
Baseline model: age, sex, tumor site, metastatic status, ECOG-PS, modified G8	0.83 [0.82; 0.85]	-	-
Baseline model + **GPS**	0.85 [0.84; 0.87]	0.07 [0.01; 0.13]	0.03 [-0.01; 0.10]
Baseline model + **mGPS**	0.85 [0.83; 0.87]	0.02 [-0.04; 0.11]	0.03 [-0.02; 0.10]
Baseline model + **CRP**	0.85 [0.83; 0.86]	0.05 [-0.04; 0.13]	0.01 [-0.03; 0.09]
Baseline model + **albumin**	0.84 [0.83; 0.86]	0.03 [-0.04; 0.12]	0.04 [-0.03; 0.09]
Baseline model + **CRP/albumin ratio**	0.85 [0.83; 0.87]	0.08 [0.02; 0.16]	0.02 [-0.03; 0.08]

ECOG-PS: Eastern Cooperative Oncology Group-Performance Status; CRP: C-reactive protein; GPS: Glasgow Prognostic Score; mGPS: modified Glasgow Prognostic Score; NRI: net reclassification improvement; CI: confidence interval.

Table S4: Multivariable analysis of GPS, mGPS, CRP, albumin and CRP/albumin ratio with regard to one-year mortality in the subgroup of patients with metastatic cancer (n=666)

**Models**	**Adjusted Hazard Ratio [95%CI]**	***P***
Normal G8 score		
GPS 0	1	
GPS 1	6.83 [1.83; 25.47]	0.004
GPS 2	23.16 [5.09; 105.42]	<0.001
Abnormal G8 score		
GPS 0	1	
GPS 1	1.98 [1.28; 3.07]	0.002
GPS 2	3.83 [2.56; 5.74]	<0.001
Normal G8 score		
mGPS 0	1	
mGPS 1	3.60 [1.15; 11.33]	0.03
mGPS 2	12.61 [3.49; 45.56]	<0.001
Abnormal G8 score		
mGPS 0	1	
mGPS 1	1.58 [1.04; 2.40]	0.03
mGPS 2	3.08 [2.18; 4.33]	<0.001
Normal G8 score		
CRP ≤10 mg/L	1	
CRP >10 mg/L	4.80 [1.72; 13.40]	0.003
Abnormal G8 score		
CRP ≤10 mg/L	1	
CRP >10 mg/L	2.46 [1.78; 3.41]	<0.001
Normal G8 score		
Albumin ≥35 g/L	1	
Albumin <35 g/L	5.37 [1.98; 14.56]	0.001
Abnormal G8 score		
Albumin ≥35 g/L	1	
Albumin <35 g/L	2.45 [1.83; 3.28]	<0.001
Normal G8 score		
CRP/albumin ratio ≤0.215	1	
CRP/albumin ratio >0.215	12.37 [2.79; 54.93]	0.001
Abnormal G8 score		
CRP/albumin ratio ≤0.215	1	
CRP/albumin ratio >0.215	2.56 [1.78; 3.67]	<0.001

Hazard ratios and p-values correspond to multivariable analysis models adjusted for age, sex, tumor site, metastatic status, ECOG-PS (Eastern Cooperative Oncology Group-Performance Status), G8 frailty screening score, with a term for the interaction between G8 and the biomarker (one model per biomarker, with each biomarker added singly to the baseline clinical model).
GPS: Glasgow Prognostic Score; mGPS: modified Glasgow Prognostic Score; CRP: C-reactive protein; CI: confidence interval.

Table S5: Discriminant power of models with GPS, mGPS, CRP, albumin and CRP/albumin ratio with regard to one-year mortality in metastatic cancer subgroup (n=666)

Model	Harrell's C [95%CI]	NRI+ [95%CI]	NRI- [95%CI]
Baseline model: age, sex, tumor site, metastatic status, ECOG-PS, G8	0.78 [0.76; 0.81]	-	-
Baseline model + **GPS**	0.81 [0.79; 0.83]	0.04 [-0.04; 0.13]	0.11 [-0.01; 0.22]
Baseline model + **mGPS**	0.81 [0.78; 0.83]	-0.01 [-0.08; 0.09]	0.18 [0.01; 0.23]
Baseline model + **CRP**	0.80 [0.77; 0.82]	0.04 [-0.06; 0.13]	0.12 [-0.04; 0.20]
Baseline model + **albumin**	0.80 [0.77; 0.82]	-0.01 [-0.09; 0.12]	0.15 [-0.02; 0.22]
Baseline model + **CRP/albumin ratio**	0.80 [0.77; 0.82]	0.10 [-0.03; 0.18]	0.06 [-0.06; 0.21]

ECOG-PS: Eastern Cooperative Oncology Group-Performance Status; CRP: C-reactive protein; GPS: Glasgow Prognostic Score; mGPS: modified Glasgow Prognostic Score; NRI: net reclassification improvement; CI: confidence interval.
